# SIRT3-Mediated Mitochondrial Regulation and Driver Tissues in Systemic Aging

**DOI:** 10.3390/genes16121497

**Published:** 2025-12-15

**Authors:** Kate Šešelja, Ena Šimunić, Sandra Sobočanec, Iva I. Podgorski, Marija Pinterić, Marijana Popović Hadžija, Tihomir Balog, Robert Belužić

**Affiliations:** Laboratory for Metabolism and Aging, Department of Molecular Medicine, Ruđer Bošković Institute, Bijenička Cesta 54, 10000 Zagreb, Croatia; kate.seselja@irb.hr (K.Š.); ena.simunic@irb.hr (E.Š.); ssoboc@irb.hr (S.S.); iva.skrinjar@irb.hr (I.I.P.); marija.pinteric@irb.hr (M.P.); mhadzija@irb.hr (M.P.H.); balog@irb.hr (T.B.)

**Keywords:** SIRT3, mitochondrial acetylation, systemic aging, senescence, aging drivers, extracellular vesicles, NAD^+^ metabolism, inflammaging, sex differences

## Abstract

Mitochondrial dysfunction is a defining hallmark of aging that connects redox imbalance, metabolic decline, and inflammatory signaling across organ systems. The mitochondrial deacetylase SIRT3 preserves oxidative metabolism and proteostasis, yet its age-related decline transforms metabolically demanding organs into sources of pro-senescent cues. This review synthesizes evidence showing how SIRT3 loss in select “driver tissues”—notably liver, adipose tissue, vascular endothelium, bone-marrow macrophages, and ovary—initiates systemic aging through the release of cytokines, oxidized metabolites, and extracellular vesicles. We discuss molecular routes and mediators of senescence propagation, including the senescence-associated secretory phenotype (SASP), mitochondrial-derived vesicles, and circulating mitochondrial DNA, as well as sex-specific modulation of SIRT3 by hormonal and intrinsic factors. By integrating multi-tissue and sex-dependent data, we outline a framework in which SIRT3 activity defines the mitochondrial threshold separating local adaptation from systemic aging spread. Targeting SIRT3 and its NAD^+^-dependent network may offer a unified strategy to restore mitochondrial quality, dampen chronic inflammation, and therefore recalibrate the aging dynamics of an organism.

## 1. Introduction

Aging is now recognized as a progressive, systemic process rather than a simple accumulation of autonomous cellular defects. Increasing evidence indicates that dysfunction originating in specific tissues can propagate throughout the body through secreted factors, extracellular vesicles and metabolites, establishing a network of inter-organ communication that shapes organismal aging dynamics [1,2,3]. Within this framework, mitochondria act as both sensors and generators of cellular stress, integrating redox, metabolic, and signaling pathways that determine the balance between survival, adaptation, and senescence [3,4]. Mitochondrial dysfunction represents one of the twelve recognized hallmarks of aging [1]. Through shared metabolic and redox pathways, it interacts closely with other hallmarks, including, but not limited to, loss of proteostasis, genomic instability, altered intercellular communication, stem-cell exhaustion, and chronic inflammation.

Among mitochondrial regulatory proteins, Sirtuin 3 (SIRT3) has emerged as a key metabolic checkpoint. SIRT3 is a mitochondrial NAD^+^-dependent deacetylase that deacetylates and activates several enzymes involved in oxidative metabolism and antioxidant defense, including superoxide dismutase 2 (SOD2), isocitrate dehydrogenase 2 (IDH2), and components of the electron-transport chain [5,6,7,8]. Through these actions, SIRT3 preserves mitochondrial redox homeostasis, supports ATP production, and prevents oxidative protein damage [5,7,9]. Loss of SIRT3 activity—through chronological aging, HFD, NAD^+^ depletion, or genotoxic stress—leads to mitochondrial protein hyper-acetylation, increased reactive oxygen species (ROS) formation, decreased mitophagy, and metabolic inflexibility [8,10,11]. These alterations push cells toward a senescent phenotype, characterized by irreversible growth arrest and the secretion of a complex array of pro-inflammatory and pro-fibrotic mediators known as the senescence-associated secretory phenotype (SASP) [12,13]. Importantly, SIRT3 down-regulation has been documented in multiple aging tissues—liver, adipose tissue, cardiac muscle, bone, and ovary—each showing increased oxidative damage and loss of mitochondrial efficiency [8,14,15,16,17]. Such observations raise a fundamental question: does SIRT3 loss drive metabolically demanding tissues to become initiating sites of organismal aging?

These tissues, due to their metabolic load and endocrine connectivity, may operate as “driver tissues” of systemic aging. When mitochondrial regulation collapses locally, senescent cells accumulate and emit signals like cytokines, growth factors, lipids, and extracellular vesicles (EVs) that propagate mitochondrial stress and inflammation to distant organs [18,19,20]. Consequently, mitochondrial imbalance within one tissue can initiate systemic signaling cascades that amplify oxidative and inflammatory stress, accelerating organismal aging.

To evaluate how current evidence supports this framework, we examine the mechanisms underlying inter-tissue transmission of senescence, the defining traits of SIRT3-dependent driver organs, the influence of sex-specific signaling, and the therapeutic potential of modulating SIRT3 and NAD^+^ metabolism. Together, these themes provide a conceptual basis for understanding how mitochondrial function in select tissues shapes organismal aging and how its restoration through SIRT3 targeting might mitigate systemic decline.

## 2. Aging Driver Tissues Are SIRT3 Dependent

Mitochondria are central orchestrators of the aging process, as their dysfunction contributes to nearly all recognized hallmarks of aging—including genomic instability [21], loss of proteostasis [22], stem-cell exhaustion [23], and chronic inflammation [24], largely through dysregulated redox balance, NAD^+^ depletion [10,25], and release of pro-inflammatory signals [1,26,27]. Aging is not uniformly distributed across all tissues: the extent to which a tissue relies on mitochondrial integrity therefore shapes not only its intrinsic aging rate but also its capacity to influence aging systemically. We define aging driver tissues as organs or cell compartments that (i) exhibit high dependency on mitochondrial quality control and redox homeostasis, (ii) senesce early or under modest metabolic/NAD^+^ stress, and (iii) export endocrine-like aging signals, primarily soluble SASP factors and/or EV cargo, that can induce senescence in distant tissues. Among the molecular regulators that influence these features, SIRT3 stands out as one of the most conserved and experimentally validated controllers of mitochondrial homeostasis. Unlike signaling factors that act indirectly through cytosolic stress cascades, SIRT3 directly deacetylates multiple mitochondrial enzymes involved in the tricarboxylic acid cycle, fatty-acid β-oxidation, and the electron-transport chain, thereby sustaining oxidative metabolism and antioxidant capacity. This direct regulatory role positions SIRT3 loss as a central driver of mitochondrial dysfunction, simultaneously amplifying ROS generation, compromising NAD^+^-coupled metabolic efficiency, and lowering the cellular threshold for stress-induced senescence. This establishes a conserved axis through which mitochondrial imbalance accelerates organismal aging. While other regulators such as AMPK, PGC-1α, mTOR, and NAD^+^ salvage enzymes (NAMPT, CD38) also modulate mitochondrial metabolism, these pathways frequently converge on SIRT3. AMPK and PGC-1α activation increase SIRT3 transcription [28], and the NAD^+^ salvage pathway provides the essential co-substrate for its deacetylase activity [10]. In turn, SIRT3 regulates oxidative stress responses and redox balance by deacetylating and activating SOD2 and IDH2 [5,6]. Other organs, such as skeletal muscle, brain, and heart, also accumulate damaged or senescent cells, yet their contribution to systemic decline is more likely cell-autonomous or local rather than via broad endocrine-like secretory loops [29,30]. Thus, the extent of a tissue’s dependence on SIRT3-mediated mitochondrial integrity determines its potential to initiate and propagate organismal aging.

## 3. Cell Senescence

### 3.1. Local Senescence Transmission

While the main characteristics of senescent cells are proliferation arrest and resistance to apoptosis, these cells remain highly metabolically active. They secrete a distinct set of cytokines, chemokines, growth factors, proteases, bioactive lipids, and extracellular vesicles that alter intercellular communication. This secretory profile is known as senescence-associated secretory phenotype (SASP), a term coined by Campisi et al. in the early 2000s [12]. In early development, SASP functions as a programmed, transient signaling mechanism that shapes tissues through paracrine cues, guiding morphogenesis and differentiation [31,32]. Here, senescence occurs as a programmed and transient process, often called developmental senescence, distinct from canonical stress-induced forms such as oncogene-induced senescence (OIS) or replicative senescence (RS). This developmental form is typically p21Cip1-dependent, independent of DNA damage and p16Ink4a, and is accompanied by a regulated SASP that includes TGF-β, IL-6, and matrix metalloproteinases. Rather than being inflammatory, this developmental SASP acts through paracrine signaling to guide morphogenesis, tissue remodeling, and differentiation, after which senescent cells are swiftly cleared by macrophages. By contrast, senescence in aging tissues predominantly reflects replicative senescence (RS) due to telomere shortening [33,34,35], oncogene-induced senescence (OIS) [36,37] triggered by aberrant proliferation signals [38] and stress-induced premature senescence (SIPS) from oxidative, mitochondrial, or metabolic stress [39,40,41,42,43,44,45]. In these contexts, senescent cells accumulate because immune clearance becomes inefficient. As senescent cells accumulate locally within tissues, their SASP exerts paracrine effects on neighboring stromal, epithelial, endothelial, and immune cells, thereby altering tissue architecture and homeostasis. In the early stages of senescence, the SASP is characterized by immunosuppressive and tissue-remodeling factors, including TGF-β, VEGF, PDGF, and MMPs, which promote matrix deposition, angiogenesis, and transient immune evasion [46,47,48,49]. This phase can support wound repair and tissue regeneration by recruiting macrophages and fibroblasts. However, when senescent cells persist due to defective immune clearance, the SASP undergoes a temporal switch toward a pro-inflammatory phenotype, enriched in IL-6, IL-1β, CXCL1, and TNF-α, which sustains chronic inflammation and drives fibrosis and tissue dysfunction [13,47,50]. This chronic SASP reinforces paracrine senescence, propagating senescent state across tissue microenvironments [49,51]. Locally, accumulated senescent fibroblasts and endothelial cells contribute to extracellular matrix degradation and vascular stiffening, while senescent immune cells impair immune surveillance, amplifying low-grade inflammaging [52,53]. In adipose tissue, for example, persistent SASP factors such as IL-6 and MCP-1 disrupt insulin signaling and promote macrophage infiltration, linking senescent cell buildup to metabolic dysfunction and insulin resistance [54,55,56]. Similarly, in the liver, senescent stellate and endothelial cells secrete TGF-β and MMPs that drive fibrogenesis and cirrhotic remodeling [57,58].

### 3.2. Systemic Spread of Aging Signals

While paracrine SASP mediates local tissue remodeling, mounting evidence shows that senescence also spreads systemically, establishing a body-wide network of pro-aging signals. Senescent cells release soluble factors and extracellular vesicles (EVs) that enter the circulation, reprogramming distant cells toward a senescent or inflammatory phenotype. Transplantation of senescent fibroblasts into young mice is sufficient to induce frailty, reduce physical performance, and trigger senescence markers in multiple distant tissues [18]. Likewise, heterochronic parabiosis, i.e., joining the circulations of young and old animals, rapidly imposes aging phenotypes on the young partner, including increased p16Ink4a expression, DNA damage, and mitochondrial dysfunction across multiple organs [19]. These observations strongly suggest that circulating senescence-associated factors can act as endocrine drivers of systemic aging. Recent mechanistic studies have identified specific mediators of this long-range propagation. Hepatocyte senescence, for instance, triggers renal and cardiac senescence through TGF-β-dependent signaling, and pharmacological inhibition of TGF-β in vivo prevents transmission of senescence to extrahepatic organs [20]. Furthermore, bone marrow-derived macrophage EVs from aged mice carry miRNAs and oxidized lipids that induce senescence in endothelial and epithelial cells [59]. Together, these findings support a model in which senescence spreads through both humoral factors (cytokines, chemokines, hormones) and vesicular routes (exosomes, microvesicles), forming an “aging signaling network” reminiscent of endocrine regulation.

Beyond soluble SASP factors and EVs, several complementary routes of senescence transmission have been identified. Damage-associated molecular patterns (DAMPs) released from stressed or dying cells, including cell-free mitochondrial DNA (cf-mtDNA), whole mitochondria, oxidized nucleotides, and mitochondrial peptides [60,61,62] act as potent activators of innate immunity through TLR9 and cGAS–STING pathways, promoting secondary senescence in distant tissues. Circulating cf-mtDNA levels increase with age and correlate with inflammatory cytokines and frailty, indicating that mitochondrial debris functions as a long-range pro-aging signal [63,64]. In addition, intercellular mitochondrial transfer—through tunneling nanotubes, microvesicles, or cell fusion events—has emerged as another layer of cross-tissue communication. While primarily studied in metabolic rescue and cancer contexts, published data show that mitochondria released from stressed or senescent cells can integrate into recipient cells and alter their redox and inflammatory status [65,66]. Such exchanges may propagate mitochondrial dysfunction or restore energy homeostasis depending on context, further emphasizing mitochondria as both targets and source vectors of systemic aging. Together, these diverse mechanisms—SASP/EV signaling, DAMP release, cf-mtDNA, parabiosis and senescent-cell transplantation models, and intercellular mitochondrial transfer—highlight that senescence spreads through a multimodal communication network. Each route ultimately converges on mitochondrial stress responses and innate immune activation, reinforcing the view that systemic aging is a coordinated, mitochondria-centered process rather than a purely cell-autonomous decline.

This concept defines aging as an inter-organ signaling process rather than a purely cell-intrinsic decline. The composition and persistence of circulating SASP and EV cargo thus determine whether an organism maintains tissue homeostasis or enters a self-amplifying pro-senescent state [67,68,69,70]. The systemic propagation of senescence is tightly linked to mitochondrial function, which is considered to affect both senescence onset and organismal aging. Through its control of cellular redox balance, NAD^+^ metabolism [71], and extracellular-vesicle (EV) biogenesis, mitochondrial integrity dictates not only whether a cell enters the senescent state but also how effectively this state is communicated to distant tissues. Perturbations in mitochondrial function elevate ROS levels, deplete NAD^+^, and promote the release of mitochondria-derived EVs enriched in oxidized proteins and mtDNA [72,73]. These factors collectively amplify senescence-associated inflammatory signaling and facilitate inter-organ transmission of aging signals.

## 4. SIRT3 and the Mitochondrial Control of Senescence Propagation

SIRT3 is directly involved in mitochondrial quality-control (QC) systems. By deacetylating factors that regulate mitochondrial ROS detoxification, protein homeostasis, and metabolic flux, SIRT3 supports conditions that favor mitophagy and limit accumulation of damaged mitochondria. These QC effects complement its metabolic effects and help maintain a functional mitochondrial pool during aging. Given its central role in mitochondrial quality control, SIRT3′s functions imply that it may influence how senescence propagates beyond its tissue of origin. Biochemically, SIRT3 promotes mitochondrial enzyme activation by removing ε-acetyl-lysine modifications in an NAD^+^-dependent reaction—thereby increasing catalytic turnover and substrate affinity of multiple metabolic enzymes, including SOD_2_, IDH2, LCAD and electron-transport chain components [5,7]. A detailed discussion of the structural enzymology and full acetylome remodeling by SIRT3 falls outside the scope of this review. For interested readers, we refer to the comprehensive reviews by Zhang et al. [74] and Wei et al. [75]. Furthermore, although SIRT3 is well studied for its mitochondrial functions, few studies have examined its nuclear deacetylation activity in the context of cellular senescence, particularly within tissues that could act as systemic aging drivers. To date, the SIRT3–heterochromatin study in human mesenchymal stem cells stands as the clearest example linking SIRT3′s nuclear role to senescence in a stem-cell compartment with broad organismal impact. SIRT3 is essential for maintaining heterochromatin integrity in hMSCs—a progenitor population whose dysfunction can propagate aging signals across multiple tissues. Loss of SIRT3 leads to chromatin decompaction, detachment of lamina-associated domains, derepression of repetitive elements, and increased DNA-damage signaling, together activating a strong senescence program. Restoring SIRT3 reverses these defects, underscoring its key nuclear role in safeguarding chromatin stability and highlighting the possibility that SIRT3 loss in stem-cell driver tissues contributes directly to systemic aging [76,77].

**Figure 1:**
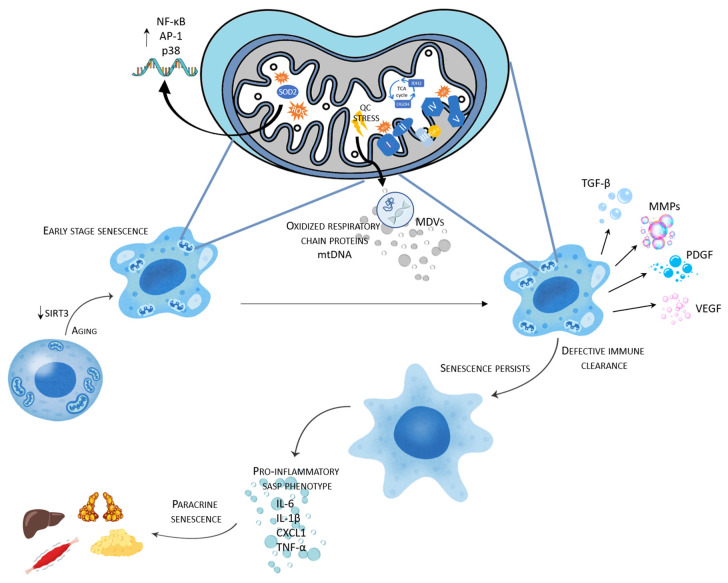
Decreased SIRT3 activity leads to elevated mitochondrial ROS, defective oxidative phosphorylation and accumulation of hyperacetylated, dysfunctional enzymes, thus favoring senescence induction and intensifying the propagation of senescence via secretory and vesicular routes. Main SIRT3 targets in mitochondria are labeled with blue color.

Decreased SIRT3 activity, either through age-related NAD^+^ depletion, metabolic overload or genetic loss, leads to elevated mitochondrial ROS, defective oxidative phosphorylation and accumulation of acetylated, dysfunctional enzymes [11,14,78]. These changes not only favor senescence induction but also intensify the propagation of senescence via secretory and vesicular routes (Figure 1). SIRT3 loss enhances mitochondrial ROS and activates the NF-κB and p38 MAPK pathways that drive SASP gene expression [79,80]. Mechanistically, SIRT3 restrains ROS by deacetylating key mitochondrial antioxidants, most notably SOD2; when SIRT3 activity falls, SOD2 becomes hyper-acetylated, ROS levels increase, and redox-sensitive transcription (e.g., NF-κB/AP-1) and stress kinases (e.g., p38) are engaged [5,9,79,80]. In parallel, SIRT3 decline impairs oxidative phosphorylation and increases global mitochondrial protein acetylation [8], promoting a senescence-prone state. Beyond transcriptional programming, mitochondrial stress modulates secretory and vesicular outputs. Under mitochondrial quality-control stress, cells selectively load oxidized respiratory-chain proteins and mtDNA into extracellular vesicles via mitochondria-derived vesicles (MDVs)—cargo capable of activating innate immune sensors in recipient cells and thereby propagating inflammatory/senescent phenotypes [72]. Consistent with these mechanisms in vivo, SIRT3^−^/^−^ hearts show NF-κB/AP-1 activation with spontaneous fibrosis [79] and SIRT3 deficiency accelerates cardiac hypertrophy/fibrosis under stress, in part via ROS-dependent signaling [81]. HFD reduces (HFD) SIRT3 and produces mitochondrial hyper-acetylation with oxidative metabolic defects [8], generating a milieu that can potentiate SASP programs and endocrine-like inflammatory output.

In line with this, pharmacologic or genetic restoration of SIRT3 activity improves mitochondrial respiration and lowers ROS in a SIRT3-dependent manner [79], while small-molecule SIRT3 activation reduces fibrosis and inflammatory signaling in the heart [82]. Altogether, these observations link tissue SIRT3 status to both local and systemic aspects of aging, a relationship we next explore across several organs, beginning with the liver.

### 4.1. Liver—Metabolic and Endocrine Driver

The proper functioning of the liver as a central metabolic organ is essential for the maintenance of energy balance and overall health. Aging is associated with increased hepatic fat accumulation, impaired response to oxidative stress, and diminished regenerative capacity, all of which contribute to the onset of various age-related diseases such as type 2 diabetes, non-alcoholic fatty liver disease (NAFLD), cardiovascular, and neurodegenerative disorders. Using acute liver senescence as a model, it has been demonstrated that hepatocytes undergoing cellular deterioration drive dysfunction in distal tissues [20], suggesting that the liver is not merely a passive target of age-related decline but can actively contribute to systemic aging. Since it has also been shown that hepatocytes can transmit signals to distal tissues through extracellular vesicles (EVs) [83], the liver emerges as a strong candidate for a driver tissue of age-related systemic decline. Considering that mitochondrial dysfunction is one of the central mechanisms underlying age-associated changes, we hypothesize that loss of SIRT3, a major mitochondrial deacetylase, could trigger mitochondrial dysfunction in the liver, leading to the release of SASP factors and EVs that transmit aging signals to other organs. SIRT3 expression in the liver is dynamically regulated in response to nutritional stress, and studies conducted on animal models have shown that its levels are modulated by HFD exposure. More precisely, in animal models, short-term exposure to an HFD induces an initial increase in hepatic SIRT3 levels, whereas prolonged HFD feeding leads to a significant downregulation at both mRNA and protein levels, accompanied by elevated global mitochondrial protein acetylation. Under prolonged HFD exposure, SIRT3 knockout (SIRT3KO) mice exhibited accelerated obesity, impaired glucose homeostasis, and insulin resistance, along with pronounced hepatic steatohepatitis and fibrosis, compared to WT controls. When an adenovirus was used to increase SIRT3 expression, it reversed these negative effects. Specifically, hepatic triglyceride levels were 50% lower in WT mice injected with a SIRT3-expressing adenovirus than in control mice [8]. Taken together, these results indicate that hepatic SIRT3 deficiency under nutritional stress can impair systemic metabolic health and promote aging-like phenotypes. Consistent with findings in animal models, SIRT3 expression was found to be downregulated at both mRNA and protein levels in the livers of NAFLD patients, compared to healthy controls [84]. In contrast, fasting has been shown to have the opposite effect. Hepatic SIRT3 expression, which is low under basal conditions, is strongly induced after 24 h of fasting in mouse models [7]. Using quantitative mass spectrometry to analyze the liver mitochondrial acetyl-proteome under caloric restriction in WT and SIRT3 KO mice, it was shown that caloric restriction induces extensive reprogramming of mitochondrial protein acetylation and that SIRT3 is an important regulator of caloric restriction adaptation [85]. This reciprocal regulation suggests that SIRT3 acts as a metabolic sensor that responds to nutrient availability, promoting mitochondrial adaptation during energy scarcity while being suppressed under chronic nutrient excess. Another important finding highlighting the role of SIRT3 in NAFLD and overall metabolic health comes from a study analyzing samples from individuals with NAFLD. Through various analytical approaches, a single nucleotide polymorphism (SNP) in the SIRT3 gene was identified as being significantly associated with increased susceptibility to metabolic syndrome [8]. Further analyses revealed that this polymorphism leads to a mutation in the SIRT3 gene that reduces its enzymatic activity, which may explain the higher incidence of metabolic syndrome observed in individuals carrying this genetic variant. Intriguingly, a genetic polymorphism that enhances SIRT3 function has been associated with increased longevity in humans, as demonstrated in the TRELONG study, which followed a systematically selected cohort of elderly participants from Treviso, Italy [86]. Several studies have identified different mechanisms through which hepatic SIRT3 may influence the development and progression of NAFLD. For instance, SIRT3 has been shown to promote fatty acid β oxidation by deacetylating key enzymes in this pathway, such as long-chain acyl-CoA dehydrogenase (LCAD) [8]. Additionally, another study confirmed that SIRT3 directly deacetylates LCAD to enhance fatty acid β-oxidation), as well as inhibits stearoyl-CoA desaturase 1 (SCD1), thereby suppressing lipogenesis and ameliorating lipotoxicity in hepatocytes [87]. The authors also provided evidence that SIRT3 promotes macroautophagy and chaperone-mediated autophagy to prevent hepatic lipid accumulation. In addition, a study using WT and SIRT3KO mice fed an HFD, as well as SIRT3-knockdown human Huh-7 cells, demonstrated that SIRT3 deficiency in the liver increases nuclear LIPIN1 and hypoxia-inducible factor 1-alpha (HIF-1α) levels, promoting lipid accumulation [88]. This was accompanied by upregulation of lipid uptake proteins, including CD36 and the VLDL receptor, an effect modulated by nuclear factor erythroid 2-related factor 2 (Nrf2) signaling. Moreover, it has been reported that Nrf2-dependent upregulation of SIRT3 protects hepatocytes from ER stress-induced injury and may prevent further progression of liver disease [89].

In addition to ectopic lipid accumulation in hepatocytes and subsequent lipotoxicity, one of the major hallmarks of NAFLD is elevated oxidative stress, which contributes to hepatic aging and associated functional decline. Both in vitro and in vivo studies have shown that SIRT3 levels are reduced in hepatocytes subjected to oxidative injury [90]. By enhancing reactive oxygen species (ROS) detoxification and maintaining mitochondrial function, SIRT3 plays a central role in protecting hepatocytes from oxidative stress. At the mechanistic level, peroxisome proliferator-activated receptor gamma coactivator 1-alpha (PGC-1α) regulates SIRT3 expression in hepatocytes, with SIRT3 acting as a downstream mediator of PGC-1α-induced control of ROS production and mitochondrial biogenesis, thereby inhibiting ROS formation [28]. Elevated hepatic oxidative stress may also have systemic consequences, as damaged hepatocytes can release signaling molecules and EVs that influence distal tissues [91], suggesting that SIRT3 could have a role in linking hepatic oxidative stress to whole-body aging.

To summarize, findings from these multiple studies point to the importance of SIRT3 in metabolic disorders such as NAFLD, which has recently been redefined as metabolic dysfunction-associated fatty liver disease (MAFLD) to better reflect the fact that it is a systemic disorder rather than a liver-limited condition [92]. In this context, MAFLD can be considered not only a metabolic disorder but also a manifestation of systemic aging processes driven by chronic nutrient overload and mitochondrial dysfunction. It is evident that loss of hepatic SIRT3 leads to a more severe MAFLD phenotype through multiple mechanisms, including impaired mitochondrial function, increased oxidative and endoplasmic reticulum stress, reduced fatty acid β-oxidation, and enhanced lipogenesis. In addition, loss of hepatic SIRT3 contributes to the development of the metabolic syndrome at the whole organism level, as shown in animal models, highlighting SIRT3 as a potentially relevant factor not only for liver function but also in the acceleration of systemic aging processes.

With regard to whether hepatic SIRT3 expression changes during aging, existing studies report inconsistent findings. For instance, no significant age-related differences in either mRNA or protein levels of SIRT3 have been observed in the livers of young versus old rats [17]. In contrast, another study reported that SIRT3 protein levels were markedly reduced in the mitochondrial fraction of livers from 20-month-old mice [93]. It is possible that variables such as strain, age, sex, the specific liver fraction analyzed (whole tissue vs. isolated mitochondria), or dietary background may account for the observed discrepancies. Even when SIRT3 protein levels remain unchanged, its activity can be significantly reduced, as observed in the livers of HFD-fed mice, where SIRT3 activity decreased by 38% regardless of stable expression [94].

Despite the heterogeneous data regarding hepatic SIRT3 expression during aging, there is clear evidence that its enzymatic activity declines with age, suggesting a progressive loss of functional capacity [10]. Enzymatic activity of SIRT3 is tightly dependent on intracellular NAD^+^ availability. A growing body of research indicates that NAD^+^ levels decline with aging, contributing to mitochondrial dysfunction and metabolic imbalance. Strategies aimed at boosting NAD^+^ levels, ranging from simple supplementation with its precursors to pharmacological modulation of NAD^+^-metabolizing enzymes, have been widely explored as potential approaches to enhance sirtuin activity, restore mitochondrial function, and treat various age-related conditions, including metabolic, cardiovascular, and neurodegenerative disorders [95].

The mechanisms underlying the age-associated decline in NAD^+^ levels have been investigated, revealing that tissue NAD^+^ concentrations sharply decrease with age, contributing to mitochondrial dysfunction and metabolic impairment [10]. A sharp decline in tissue NAD^+^ levels was observed in aging mice, which positively correlated with a decrease in mitochondrial function. Furthermore, the study revealed that CD38 expression increases with age across multiple tissues, including the liver, and plays a central role in mediating the age-related depletion of NAD^+^. In the liver of 1-year-old CD38KO mice, increased NAD^+^ availability enhanced SIRT3 activity, resulting in improved mitochondrial function and better glucose regulation. When both CD38 and SIRT3 were deleted (CD38/SIRT3 double knockout), these beneficial effects disappeared and mitochondrial function and glucose tolerance phenotype returned to levels observed in WT animals. Collectively, these findings demonstrate that an increase in NAD^+^ alone is not sufficient, but active SIRT3 is required to utilize NAD^+^ and maintain mitochondrial health during aging. This study is consistent with the hypothesis discussed in our review article and suggests that, with aging, the liver progressively loses the protective function of SIRT3 due to cofactor (NAD^+^) depletion, thereby contributing to increased susceptibility to cellular senescence.

Downregulation of SIRT3 not only leads to disruption of metabolic health, as discussed previously, but also compromises the regenerative capacity of the liver [96]. To investigate the role of SIRT3 in liver regeneration, the authors generated a hepatocyte-specific SIRT3KO model and demonstrated that these mice exhibited a markedly reduced regenerative capacity following partial hepatectomy. Loss of SIRT3 resulted in significant impairments in multiple parameters of liver regeneration, as evidenced by decreases in the liver-to-body weight ratio, hepatocyte proliferation, and hepatic energy metabolites (triglycerides, NAD^+^, NADH, and ATP). This highlights that SIRT3 is not only essential for maintaining hepatic metabolic health but also plays a central role in liver regeneration. Thus, the gradual loss of SIRT3 function during aging may lead not only to metabolic decline, but also to a reduced capacity for hepatic regeneration, which can actively influence the aging progression of distal tissues.

In summary, it is evident that hepatic SIRT3 plays an important role in proper liver functioning, and age-related decline or downregulation of SIRT3 activity in response to nutritional status compromises these protective functions, contributing to metabolic dysfunction, impaired liver regeneration, and potentially promoting systemic aging. Therefore, preserving or restoring SIRT3 activity, whether through nutritional interventions, direct pharmacological modulation or NAD^+^-boosting strategies, could represent a promising approach to prevent the progression of MAFLD, delay liver aging and counteract systemic age-related decline. In other words, exploring how targeted modulation of SIRT3 in the liver influences systemic health and longevity could help further advance our understanding of this relevant research field. In particular, future studies should aim to elucidate the precise signaling mechanisms by which hepatic SIRT3 communicates with other organs to regulate systemic metabolism and aging. It remains to be determined how hepatic SIRT3-dependent changes influence the composition and signaling properties of EVs released from hepatocytes, and whether these vesicles contribute to the propagation of senescence-associated phenotypes across other tissues.

### 4.2. Adipose Tissue (WAT and BAT)—Metabolic–Inflammatory Axis

White adipose tissue (WAT) is the most abundant type of fat tissue in mammals, primarily functioning as an energy reservoir that stores excess energy in the form of triglycerides. It is widely distributed throughout the body, with major depots located subcutaneously and viscerally around internal organs. It also serves as an active endocrine organ regulating metabolism, insulin sensitivity and inflammation. Adipocytes in WAT secrete key adipokines such as leptin and adiponectin, while resident immune cells release proinflammatory cytokines including TNF-α and IL-6. During aging, both pro- and anti-inflammatory adipokine levels increase; however, the balance shifts toward proinflammatory signaling, leading to a state of chronic, low-grade inflammation [97]. Aging also alters WAT quantity, distribution, and function. Studying the interplay between aging and WAT endocrine activity in humans is challenging due to age-associated morphological remodeling of adipose tissue and the frequent coexistence of metabolic syndrome, insulin resistance, and obesity [98]. Nevertheless, these changes in WAT are a hallmark of the aging process and contribute to the systemic proinflammatory state known as inflammaging. Inflammaging is driven by several interconnected mechanisms, including cellular senescence, mitochondrial dysfunction, defective autophagy and mitophagy, inflammasome activation, and persistent DNA damage responses [99]. SIRT3 is a key regulator of many of these processes, making it a potential modulator of inflammaging. However, to the best of our knowledge, no studies have directly examined the age-related decline of SIRT3 function specifically in the context of WAT inflammaging, likely due to the relatively low basal expression of SIRT3 in this tissue. In contrast, SIRT1 is ubiquitously expressed and abundant in WAT. It has been shown to suppress NF-κB activity, reduce inflammation, and exert anti-apoptotic and anti-aging effects [100], highlighting its central role in controlling WAT inflammaging. Studies using adipocyte-specific SIRT3 KO mice report no significant metabolic alterations with aging compared to controls, suggesting that tissue-specific SIRT3 loss alone may not drive age-related metabolic changes. Nonetheless, global SIRT3 expression and activity decline with age, leading to hyperacetylation of mitochondrial enzymes [101], increased reactive oxygen species (ROS) production, and impaired fatty acid oxidation [7]. Elevated ROS serves as a potent activator of inflammatory and senescence pathways, promoting the SASP that underlies inflammaging [102]. Although basal SIRT3 levels in WAT are low, diminished SIRT3 function may still contribute to the overall systemic inflammation associated with aging. Supporting this idea, studies on SIRT3 and inflammation demonstrate that SIRT3 expression and activation are closely linked to mitochondrial bioenergetics and the regulation of macrophage inflammatory responses. Macrophages derived from SIRT3 KO mice display altered mitochondrial metabolism and redox homeostasis, accompanied by a proinflammatory phenotype characterized by NLRP3 inflammasome activation [103]. Altogether, these findings suggest that while Sirt1 is a key anti-inflammatory regulator in WAT, SIRT3 likely contributes to inflammaging by modulating mitochondrial function and redox balance. Further studies are needed to clarify the specific role of SIRT3 in age-related changes within WAT and its potential interaction with Sirt1 in maintaining adipose tissue homeostasis.

#### 4.2.1. SIRT3 in Maintaining Brown Adipose Tissue Thermogenic Capacity

Brown adipose tissue (BAT) plays a central role in energy balance, thermoregulation, and metabolic health. It is a mitochondria-rich tissue specialized for non-shivering thermogenesis, a process driven by uncoupling protein 1 (UCP1). UCP1 is an abundant protein in the mitochondrial inner membrane that induces proton leak–mediated respiration, while SIRT3 acts as a crucial regulator of the electron transport chain by deacetylating and maintaining the activity of key oxidative phosphorylation enzymes, thereby preserving mitochondrial efficiency and redox balance. Since thermogenic activation results in the uptake and oxidation of large amounts of glucose and fatty acids (FAs), it requires high oxidative metabolism, making SIRT3 a crucial regulator of BAT function. BAT expresses high levels of SIRT3 [101], and cold exposure further increases its transcript abundance [104]. Moreover, fasting SIRT3 KO mice display reduced fatty acid oxidation in BAT and intolerance to acute cold stress [7], demonstrating the importance of SIRT3 in sustaining BAT oxidative metabolism and thermogenic capacity. When BAT is activated, Ucp1 gene expression increases under the control of multiple transcription factors. A study from 2019 suggests that SIRT3 indirectly controls BAT thermogenesis by deacetylating and promoting the function of pathways upstream of UCP1, providing strong evidence that SIRT3-mediated deacetylation is fundamentally important for BAT activation [105]. In line with this, Zhang et al. generated adipose-specific SIRT3 KO mice and showed that they display defective acylcarnitine metabolism and thermogenesis in interscapular BAT (iBAT) during cold exposure. They further demonstrated that SIRT3 modulates transcription of genes involved in acylcarnitine metabolism via regulation of the hypoxia-inducible factor 1α (HIF1α)–peroxisome proliferator-activated receptor α (PPARα) signaling pathway to promote BAT thermogenesis [106]. Efficient BAT activity is important for the maintenance of metabolic homeostasis, which makes BAT a key tissue in the context of aging-related metabolic decline. During aging, BAT mass and activity decrease [107], and brown adipocytes undergo a process of whitening, characterized by increased lipid accumulation and mitochondrial loss [108]. Some studies report a marked decline in UCP1 expression with age [109], while others find little or no change [110]. Tajima et al. performed RNA-seq and mitochondrial proteomics on BAT from young (10-week-old) and aged (76-week-old) mice, revealing broad disruption of mitochondrial oxidation pathways, including fatty acid, glucose, and branched-chain amino acid oxidation, as well as TCA cycle activity, suggesting that defective fuel oxidation contributes to age-associated BAT thermogenic decline. These mitochondrial processes are closely linked to SIRT3 function, as many enzymes involved in oxidative phosphorylation and energy metabolism are known SIRT3 targets. Thus, the decline in SIRT3 expression and activity during aging contributes to mitochondrial protein hyperacetylation, impaired oxidative phosphorylation, and elevated oxidative stress in brown adipocytes. Collectively, these changes reduce UCP1-dependent thermogenesis and blunt BAT’s capacity to generate heat in response to cold or adrenergic stimulation.

#### 4.2.2. SIRT3 as a Regulator of Skeletal Muscle Metabolism and Muscle—WAT Crosstalk

Skeletal muscle is a highly metabolically active organ essential for insulin-mediated glucose uptake and lipid catabolism. It plays a major role in systemic metabolic control and whole-body energy homeostasis. Due to its high mitochondrial content, skeletal muscle exhibits robust expression of SIRT3, which supports adaptive metabolic responses to energetic stress such as exercise or fasting. Specifically, Palacios et al. demonstrated that SIRT3 is more highly expressed in soleus muscle compared to extensor digitorum longus or gastrocnemius muscle, consistent with the distinct metabolic requirements of different muscle types. Additionally, they found that SIRT3 protein levels in skeletal muscle are sensitive to diet: caloric restriction increased SIRT3 expression, whereas a HFD decreased it [111]. Similarly, a 2022 review highlighted the importance of exercise and skeletal muscle in counteracting age-related metabolic decline, showing that regular physical activity enhances SIRT3 expression across multiple tissues and mitigates aging-induced suppression [112]. Peroxisome proliferator-activated receptor gamma coactivator-1α (PGC1-a) plays a central role in the metabolic regulation of skeletal muscle. It stimulates mitochondrial biogenesis [113], induces muscle fiber-type switch, and increases oxidative capacity in skeletal muscle cells [114]. Palacios et al. further showed that SIRT3 KO mice had decreased PGC-1α mRNA levels, and lower phosphorylation of AMPK and CREB [111]. It is known that AMPK, an important energy sensor and regulator of muscle metabolism, increases the expression of PGC1-a [115]. Furthermore, several studies show SIRT3 and AMPK form a functional axis: SIRT3 upregulation activates AMPK signaling, promoting autophagy, mitochondrial biogenesis and metabolic adaptation [116,117], while AMPK activation can also increase SIRT3 activity via upstream regulators [118]. This cross-talk provides a plausible mechanism whereby SIRT3 decline during aging could impair AMPK signaling, reducing mitochondrial turnover and metabolic flexibility in skeletal muscle. Because of its intense metabolic activity, skeletal muscle generates substantial amounts of reactive oxygen species (ROS). Kong et al. demonstrates that PGC-1α increases the expression of GPx1 and SOD2 in skeletal muscle cells, thereby suppressing ROS generation. They further showed that knockdown of endogenous SIRT3 expression increased cellular ROS levels, whereas overexpression decreased them, indicating that SIRT3 acts as suppressor of ROS formation and mediates the protective effect of PGC-1α on cellular ROS production [28]. Beyond its antioxidant role, SIRT3 is involved in mitochondrial substrate choice and metabolic flexibility of skeletal muscle, by regulating pyruvate dehydrogenase (PDH) function through deacetylation. Jing et al. reported that SIRT3 KO mice exhibited impaired glucose utilization and relied more heavily on fatty acid β-oxidation for energy, resulting in elevated acylcarnitine breakdown and ROS accumulation, which may contribute to insulin resistance over time [119]. Interestingly, these results contrast those of Palacios et al., who observed increased SIRT3 expression during fasting, while Jing et al. report decreased expression and confirm those results in SIRT3 KO mouse model. However, it is worth pointing out that fasting and specifically caloric restriction are known strategies for combating aging, which is in line with results from Palacios et al. In addition to its metabolic role, skeletal muscle functions as an endocrine organ that secretes myokines in response to contraction [120]. These signaling molecules exert autocrine, paracrine, and endocrine effects, coordinating communication between muscle and other organs. Certain myokines regulate lipid metabolism and promote the browning of WAT [121]. For instance, Knudsen et al. showed that daily intraperitoneal injections of IL-6 increased UCP1 mRNA levels in inguinal WAT [122]. This contraction-induced myokine release underscores the anti-inflammatory and anti-aging benefits of regular exercise. On the other hand, adipose tissue secretes proinflammatory adipokines during physical inactivity, contributing to metabolic diseases such as type 2 diabetes mellitus and atherosclerosis, both of which are prevalent in aging populations. Among the various cytokines involved in this muscle–adipose crosstalk, IL-6, IL-15, irisin, and myostatin play particularly important roles [123]. Irisin is a PGC-1α-dependent myokine which drives brown-fat-like development [120], suggesting the involvement of SIRT3 in muscle-adipose crosstalk. SIRT3 may indirectly drive WAT remodeling toward a more oxidative, metabolically beneficial phenotype. During aging, the decline of SIRT3 expression and activity leads to increased oxidative stress, impaired lipid metabolism, and elevated inflammatory cytokine production. These changes disrupt the healthy communication between muscle and WAT, contributing to systemic metabolic dysfunction and inflammaging.

### 4.3. Endothelial Tissue

Endothelial cells (ECs) form the primary interface between blood and parenchyma, so their senescence has outsized systemic consequences. Loss of SIRT3 in endothelium elevates mitochondrial superoxide, blunts Akt/eNOS signaling, reduces NO bioavailability, and drives endothelial dysfunction; these effects are shown in humans with obesity and in rodent hypertension models, and endothelial-specific SIRT3 knockout causes diastolic dysfunction via impaired glycolysis and angiogenesis [124,125,126]. In humans with obesity and in diet-induced obese mice, SIRT3 deficiency induces endothelial insulin resistance, increases mitochondrial ROS, and blunts insulin-stimulated vasorelaxation, linking SIRT3 loss to early vascular metabolic dysfunction [124]. Endothelial-specific SIRT3 deletion also disrupts glycolytic flux and angiogenic signaling, leading to coronary microvascular rarefaction and diastolic dysfunction in vivo [126]. Complementary findings in essential hypertension indicate that vascular SIRT3 depletion is accompanied by SOD2 hyper-acetylation, heightened oxidative stress, endothelial inflammation, and vascular remodeling, while SIRT3 overexpression reverses these phenotypes [125]. Senescent ECs secrete a SASP rich in IL-6, IL-8, MCP-1, VEGF, TGF-β, and adhesion/matrix proteins, fostering leukocyte recruitment, vascular inflammation, and pro-thrombotic remodeling [127]. Beyond soluble factors, senescent EC-derived exosomes transmit dysfunction to healthy endothelium by degrading junctional integrity and barrier function [128].

### 4.4. Bone-Marrow Macrophages

The bone-marrow macrophage niche converts mitochondrial dysfunction into a body-wide inflammatory tone. Macrophage metabolism is tightly linked to SIRT3-dependent regulation of the TCA cycle, oxidative phosphorylation, and mitochondrial antioxidant defenses. Experimental up-regulation of SIRT3 in macrophages—by estradiol or direct overexpression—restores mitochondrial membrane potential, lowers ROS, and suppresses IL-1β and TNF-α secretion, shifting polarization away from the pro-inflammatory M1 state [129]. Conversely, metabolic or diet-induced down-regulation of SIRT3 in bone-marrow-derived macrophages leads to mitochondrial protein hyper-acetylation, impaired respiration, and elevated NF-κB signaling that exacerbates systemic inflammation and metabolic dysfunction [130]. In physiological aging, bone-marrow-derived macrophages accumulate mitochondrial damage and adopt a senescence-like transcriptional program. In line with this, SIRT3 overexpression in myeloid cells diminishes inflammatory injury in endotoxin-induced acute lung injury, consistent with a role for SIRT3 in restraining mitochondrial stress–driven innate immune activation [131]. Moreover, pharmacological and genetic inhibition of SIRT3 in obesity models drives proinflammatory macrophage polarization by inducing mitochondrial dysfunction and excessive ROS, providing direct evidence that SIRT3 loss in macrophages can amplify systemic inflammatory tone [130]. Their extracellular vesicles (EVs) contain oxidized lipids and senescence-associated miRNAs; when transferred into young mice, these EVs induce p21Cip1 and γH2AX expression in liver, muscle, adipose tissue, bone, and brain, producing frailty and multi-organ senescence [59]. Collectively, these findings identify bone-marrow macrophages as mobile amplifiers of organismal aging—where compromised SIRT3-mediated mitochondrial control transforms the hematopoietic compartment into a circulating source of pro-inflammatory and pro-senescent signals.

### 4.5. Bone and Osteocyte Lineage

SIRT3 safeguards osteoblast and osteocyte mitochondrial integrity, ensuring energy supply for matrix maintenance. Its deficiency precipitates osteocyte senescence, elevated p16/p21, and bone frailty [15]. Aging bone also receives pro-senescent input from macrophage EVs [59], indicating reciprocal exchange between skeletal and immune compartments. Because bone marrow is a major cytokine and EV reservoir, mitochondrial dysfunction in this niche likely feeds systemic inflammation and contributes to musculoskeletal decline.

### 4.6. Ovary—Reproductive and Endocrine Roles

Reproductive tissues display exceptional sensitivity to mitochondrial quality control, reflecting their high energetic demand and the tight coupling between redox balance and hormonal output. Loss of SIRT3 in oocytes causes mitochondrial hyper-acetylation, decreased membrane potential, lower mtDNA copy number, and premature follicular atresia—hallmarks of reproductive senescence. In aged mice and human ovarian tissue, SIRT3 expression declines, leading to impaired steroidogenesis, decreased antioxidant defense, and altered granulosa-cell mitochondrial morphology [16]. Restoration of SIRT3 activity improves oocyte quality, fertilization rate, and mitochondrial respiration, underscoring its central role in female reproductive longevity [132].

Although a direct endocrine transmission of ovarian senescence has not yet been demonstrated, aged or SIRT3-deficient ovaries show altered cytokine and extracellular-vesicle release profiles, which may modulate systemic metabolic and inflammatory tone, influencing whole-body aging trajectories. Sex differences in mitochondrial regulation reinforce this connection: estrogen is suggested to enhance SIRT3 expression through ERα/PGC-1α signaling [133], improving mitochondrial respiration and antioxidant capacity, while female mice exhibit SIRT3-dependent protection against oxidative stress and superior mitochondrial homeostasis compared with males [134]. Collectively, these findings establish the ovary as both a reproductive and endocrine aging node, where mitochondrial quality and SIRT3-dependent regulation contribute to female-specific patterns of systemic aging and longevity.

### 4.7. Integration of Multi-Tissue Evidence

Collectively, these tissue-specific observations converge on a unifying framework: organs with high mitochondrial throughput and strong SIRT3 dependence act as upstream “aging drivers.” When mitochondrial acetylation control collapses, these tissues convert local metabolic stress into circulating inflammatory and vesicular cues—amplifying senescence across distant sites. Conversely, maintaining SIRT3 activity, whether by NAD^+^ repletion or direct activation, preserves mitochondrial proteostasis, restrains SASP induction, and interrupts this endocrine-like aging circuit. Thus, SIRT3 functions as a conserved gatekeeper of mitochondrial–immune communication, determining whether high-flux organs sustain systemic resilience or precipitate organism-wide senescence.

## 5. Sex-Specific Regulation of SIRT3 and Mitochondrial Aging

Beyond its tissue-specific expression, SIRT3 also exhibits context-dependent roles in aging and metabolic resilience. Notably, ovarian—but not testicular—aging depends strongly on SIRT3 [14], suggesting sex-specific regulation of mitochondrial function. Across studies, both SIRT3 expression and its downstream effects vary between sexes. Understanding this variation requires thinking in systems terms: hormonal regulation, chromosomal, epigenetic, and metabolic mechanisms together with intrinsic cellular differences form overlapping layers of control, together shaping how SIRT3 influences metabolism, stress adaptation, and aging.

### 5.1. Hormone-Dependent SIRT3 Regulation

Estrogen provides one of the clearest examples of hormonal control over SIRT3. Well known for its mitochondrial protective effects, estrogen modulates antioxidant enzymes and regulates genes involved in oxidative phosphorylation [135]. Both estrogen receptors (ERα and ERβ) localize to mitochondria in several cell types—breast cancer cells [136], neurons, and cardiomyocytes [137]—where they influence mtDNA transcription, biogenesis, and oxidative stress defenses. The presence of estrogen response elements within mtDNA [138] further demonstrates how steroid signaling extends directly into the mitochondrial genome.

Several recent studies strengthen this connection. Estradiol (E2) treatment upregulates mitochondrial SIRT3 expression in macrophages, particularly in female-derived bone marrow macrophages, increasing deacetylase activity and reducing protein acetylation [129]. This response appears sex-specific, possibly reflecting prior estrogen exposure. In inflammatory macrophages, E2-driven SIRT3 activation lowers reactive oxygen species and dampens inflammatory signaling—an elegant example of estrogen’s dual antioxidant and anti-inflammatory influence through SIRT3. The kidney illustrates the E2-SIRT3 relationship at the tissue level: male mice, which express less renal SIRT3 than females, are more susceptible to ischemia–reperfusion injury [139,140]. Overexpressing SIRT3 protects males, while deleting it eliminates the female advantage. Estradiol elevates SIRT3 levels, testosterone suppresses them. Hormonal state, therefore, directly influences mitochondrial defense capacity. SIRT3 sits at the center of this axis, emerging as a promising target for sex-specific protection against acute kidney injury. Although SIRT3 protects mitochondrial function in macrophages and kidney, its role in bone is paradoxical. Rather than preserving tissue integrity, SIRT3 activity promotes osteoclastic turnover—an effect that becomes especially pronounced under estrogen deficiency, revealing a sex-dependent mitochondrial mechanism in skeletal aging [15]. Deleting SIRT3 in young mice has little effect, but with age the absence of SIRT3 attenuates bone loss in both sexes. Normally, estrogen withdrawal upregulates SIRT3 in osteoclasts, stimulating mitochondrial activity and accelerating turnover. In this setting, SIRT3 acts as a mediator of estrogen-dependent bone loss—a potential target in postmenopausal osteoporosis, where hormonal decline and mitochondrial stress intersect.

Androgens seem to exert a comparable influence on SIRT3, though evidence remains limited. In a rat model of prostate hyperplasia, testosterone propionate–induced mitochondrial dysfunction was alleviated by SIRT3 overexpression through the AMPK/PGC-1α pathway, which stabilized mitochondrial membrane potential [141]. In cardiac and renal tissues, testosterone deficiency similarly impairs mitochondrial function and downregulates protective regulators such as SIRT3 [142]. In male Leydig cells, the connection is more direct. SIRT3 maintains redox balance, supports proliferation, and enables progesterone synthesis by suppressing ROS accumulation [143]. When SIRT3 is lost, oxidative stress rises, key transcriptional regulators such as Ad4BP/SF-1 and GATA4 decline, and steroidogenesis falters. Here, SIRT3 functions as a cornerstone of male mitochondrial and endocrine stability. Even without direct demonstrations in tissues with high oxidative demand, like muscle or liver, these findings suggest that androgens, like estrogens, modulate mitochondrial metabolism through signaling networks that converge on SIRT3.

Other hormones appear to influence SIRT3 as well, though sex-specific data are sparse. Melatonin upregulates SIRT3 to protect the heart from ischemia–reperfusion injury, reducing oxidative stress and apoptosis—but this effect has been tested only in male mice [144]. Similarly, reduced SIRT3 expression has been reported in humans with altered thyroid status, including diabetes and hypothyroidism, yet no sex-stratified analyses exist [145]. These gaps leave open the question of whether endocrine regulation of SIRT3 differs between sexes beyond the known effects of sex hormones, which is a limitation that future work must address.

### 5.2. Hormone-Independent Regulation of SIRT3

Sexual dimorphism in SIRT3 regulation extends beyond hormonal influence, reflecting intrinsic chromosomal, epigenetic, and metabolic distinctions that shape mitochondrial function. At the molecular level, SIRT3 operates downstream of the PGC-1α/ERRα complex, a master regulator of mitochondrial biogenesis and energy homeostasis [28]. Because sex hormones also affect PGC-1α, AMPK, and NAD^+^ metabolism, these regulatory systems often overlap. Distinguishing direct chromosomal effects from secondary hormonal influence is therefore challenging, but the balance of evidence suggests both are at play. Two recent studies underscore this point: absence of SIRT3 in male and female mouse embryonic fibroblasts (MEFs) show that SIRT3 regulates mitochondrial metabolism and redox balance in a distinctly sex-dependent manner, even under identical, hormone-free conditions [134,146]. Male SIRT3-deficient MEFs exhibit greater metabolic stress, enhanced glycolytic reprogramming, and oxidative vulnerability, whereas female cells display stronger basal antioxidant capacity but heightened sensitivity to genotoxic and age-related stress. This clear, cell-autonomous dimorphism demonstrates that sex-linked regulation of SIRT3 extends beyond endocrine control.

Consistent with sex-dependent effects observed in fibroblast models, in vivo neuronal studies also revealed that SIRT3 deletion selectively impaired metabolism, memory, and neuronal excitability in females, but not in males [147]. Importantly, the authors controlled for potential hormonal influences by stratifying female samples by diestrus or using sample sizes large enough to encompass all estrous phases, making it highly unlikely that the observed sex differences were driven by circulating hormone fluctuations. Moreover, because SIRT3 was selectively deleted in a subset of neurons, any changes in total cortical estrogen or hormone signaling were likely precluded. This strengthens the conclusion that female-specific neuronal vulnerability to SIRT3 loss arises from intrinsic, non-hormonal mechanisms, underscoring the importance of including both sexes in mechanistic and organismal studies of SIRT3 function.

In short, while hormones can amplify or mute SIRT3 activity, the deeper architecture of sexual dimorphism—embedded in chromosomes and epigenetic programs—appears to sustain these differences even when hormones are absent. The resulting picture is one of layered regulation: endocrine, genetic, and metabolic factors working together to maintain sex-specific mitochondrial behavior.

### 5.3. Sex-Biased Outcomes in SIRT3 Models

Differences in SIRT3 expression and regulation translate into distinct physiological outcomes across tissues, often revealing clear sex biases in metabolic stability, stress tolerance, and aging trajectories. These findings illustrate that SIRT3 does not operate as a uniform mitochondrial safeguard but as a context-dependent regulator whose impact is shaped by sex and tissue environment. As mentioned earlier, male mice remain more vulnerable to renal ischemia–reperfusion injury (IRI) because of lower baseline SIRT3 expression [140], resulting in increased mitochondrial reactive oxygen species (ROS) production and more vacuolation of tubular epithelial cells (indicative of damage). SIRT3 overexpression confers male protection [139]. Female mice, with higher baseline SIRT3, have reduced ROS and better mitochondrial fusion/OPA1 signalling in IRI, implicating sex-linked mitochondrial resilience. This pattern positions the kidney as a candidate “driver tissue” for male-biased aging, though the mechanisms linking local mitochondrial failure to systemic decline remain speculative.

In the liver, the bias persists, especially under HFD stress [148]: SIRT3 KO male mice experience pronounced hepatic lipid accumulation, reduced hepatic glucose uptake, and greater impairment of antioxidant (MnSOD) activity compared with females. Males also display stronger HFD-driven down-regulation of SIRT3 expression, increased expression of lipid-metabolic genes (e.g., PPARγ, CYP2E1, CYP4A14), and more pronounced disruption of mitochondrial Complex I–driven respiration. Conversely, female KO mice initially retain partial protection: under standard diet they maintain better mitochondrial respiration and less lipid accumulation compared to males. Yet when challenged (e.g., HFD together with ovariectomy), these females lose their advantage: mitochondrial CII-driven respiration and expression of protective lipid-homeostasis genes drop, whereas lipid damage and NAFLD-related gene expression increase [149]. Altogether, the hepatic data suggest a dual layer of sex-difference: males show higher dependence on SIRT3 under metabolic excess, and its absence produces a steeper decline in liver metabolic homeostasis; females appear more resilient under baseline conditions but reveal their vulnerability when hormonal (e.g., ovarian) support is removed, indicating that hormonal modulation can mask or delay SIRT3-related vulnerability. Unlike the kidney and liver, where males rely more heavily on SIRT3 expression for metabolic protection, neuronal, cardiovascular and skeletal systems appear to place this dependence on females. Deletion of SIRT3 in neurons leads to female-specific impairments in mitochondrial respiration, redox regulation, and cognitive performance [147]. These effects occur despite normal hormonal status, indicating intrinsic sex differences in neuronal regulation that persist independent of circulating hormones. A similar pattern emerges in the cardiovascular system: SIRT3 is essential for maintaining vascular and cardiac homeostasis, particularly in females. Its deletion leads to increased oxidative stress, disrupted nitric oxide signaling, and diastolic dysfunction in females, while males remain comparatively protected [150]. Under HFD, female KO mice show higher blood pressure, impaired coronary flow reserve, and more severe diastolic impairment than both WT and male counterparts. These findings indicate that female cardiovascular function depends more strongly on SIRT3-mediated mitochondrial and endothelial resilience, making its loss disproportionately detrimental in females. Contrary to protective effects observed in all other tissues, as noted earlier, SIRT3 mediates estrogen-dependent bone loss, with its absence mitigating age- and hormone-related bone deterioration, particularly in females [15], making it a potential target in postmenopausal osteoporosis. Reproductive tissues highlight the clearest manifestation of SIRT3′s sex-dependent roles, reflecting direct integration with endocrine and mitochondrial signaling. Female SIRT3 KO mice exhibit accelerated ovarian aging, characterized by depletion of follicular reserves, mitochondrial dysfunction, elevated oxidative stress, and diminished oocyte quality [14]. In contrast, male SIRT3 KO mice maintain normal spermatogenesis and testicular structure, indicating that the male germline is largely resilient to SIRT3 deficiency. These findings demonstrate that female reproductive tissues are markedly more dependent on SIRT3-mediated mitochondrial homeostasis, positioning SIRT3 as a critical determinant of ovarian longevity and female reproductive aging.

Viewed together, these findings outline a mosaic of sex-dependent mitochondrial vulnerabilities. Male-biased sensitivity predominates in metabolic organs such as the kidney and liver, where SIRT3 expression is lower and perhaps more strongly influenced by testosterone suppression. In contrast, female-biased dependence emerges in high-energy and hormone-responsive tissues: the brain, vasculature, bone, and ovary, where mitochondrial resilience relies heavily on SIRT3-mediated antioxidant and bioenergetic control. This pattern suggests that males are more susceptible to metabolic collapse under oxidative or nutritional stress, while females experience sharper declines in mitochondrial quality during hormonal transition or reproductive aging. Table 1 summarizes the sex-related differences associated with SIRT3 loss across major tissues and highlights the corresponding functional outcomes.

Many earlier studies relied exclusively on male models, masking these patterns. Progress now depends on sex-balanced experimental designs that manipulate both hormones and SIRT3 expression in a tissue-specific manner. The remaining questions are not trivial: do sex differences arise from divergent SIRT3 levels, substrate specificity, or compensation by related sirtuins such as SIRT4 and SIRT5? Confirming this will require studies designed from the ground up to capture sex as a biological variable—where mitochondrial biology is examined not as a neutral backdrop, but as a living system shaped by both genetics and gendered physiology. Integrative multi-omics comparisons across male and female tissues throughout aging may help resolve these questions.

## 6. Therapeutic and Translational Perspectives

Recognition that certain SIRT3-dependent organs act as systemic aging drivers refines therapeutic logic: rejuvenating or stabilizing a dominant driver tissue may recalibrate the entire aging network without global intervention. Because SIRT3 governs mitochondrial acetylation, redox balance, and extracellular vesicle (EV) and SASP output, restoring its activity within key tissues could suppress the endocrine-like dissemination of senescence and re-establish systemic homeostasis.

Senescence arises through organ-specific stressors that reflect each tissue’s physiological role. The liver, continuously exposed to metabolic flux and xenobiotics, readily develops mitochondrial hyper-acetylation, ROS accumulation, and SIRT3 loss, releasing TGF-β and mtDNA-bearing EVs that trigger secondary senescence in kidney, heart, and brain. The vascular endothelium, under constant hemodynamic and oxidative load, becomes a major SASP and EV source when SIRT3 declines; its reactivation normalizes nitric oxide signaling and reduces circulating pro-senescent vesicles. In the immune system, bone-marrow macrophages link metabolism and inflammation: SIRT3 induction restores oxidative metabolism, suppresses NLRP3 activity, and diminishes inflammatory EV release. In adipose and muscle, nutrient excess or inactivity lowers SIRT3 and β-oxidation, heightening cytokine output, whereas endurance exercise and cold adaptation physiologically boost SIRT3 via AMPK–PGC-1α signaling. Ovary and bone exemplify divergent contexts: ovarian SIRT3 activation improves oocyte quality and hormonal balance, while excessive activity in estrogen-deficient bone may accelerate resorption, emphasizing the need for tissue-specific modulation. This organ-centered view complements and extends senolytic and senomorphic approaches. Instead of removing or silencing senescent cells downstream, SIRT3 restoration targets the mitochondrial origin of senescence, potentially reducing systemic SASP load without cytotoxicity. SIRT3 activation could decrease dependence on broad senolytic regimens and their side effects, offering a precision alternative rooted in metabolic correction. Effective translation would rely on identifying true driver organs in humans, developing reliable biomarkers of tissue SIRT3 activity (acetyl-proteome or EV signatures), and delivering activators selectively to avoid off-target effects. Several small-molecule compounds illustrate how targeting SIRT3 in vulnerable driver tissues may produce system-wide benefits. Honokiol directly activates SIRT3 and improves mitochondrial respiration and oxidative-stress resistance in vivo [151]. Nicotinamide riboside enhances SIRT3 activity indirectly by increasing NAD^+^ availability and has shown protective effects in metabolically and oxidatively stressed tissues [152]. Polyphenols such as resveratrol and related derivatives can also engage SIRT3-linked pathways to support mitochondrial resilience under inflammatory or ischemic stress [153]. These examples highlight how pharmacological support of SIRT3 activity may help stabilize key driver organs—such as liver, heart, endothelium, adipose tissue, and ovary—and thereby mitigate systemic aging phenotypes.

A coordinated program combining driver-tissue targeting, NAD^+^ repletion, and senescence modulation could produce synergistic rejuvenation. Hepatic SIRT3 restoration may lower circulating TGF-β and oxidized EVs; endothelial activation could enhance vascular integrity and perfusion, while myeloid SIRT3 activation could suppress chronic inflammatory signaling and reduce the release of pro-senescent cytokines and extracellular vesicles. Restoring mitochondrial integrity in a single driver organ could recalibrate systemic aging dynamics, indicating that rejuvenation may be achievable through targeted network repair rather than global reversal.

## 7. Conclusions and Outlook

− SIRT3 links mitochondrial protein deacetylation with redox balance, metabolic efficiency, and stress resistance, positioning it as a central node in the systemic coordination of aging.
− Emerging evidence supports the existence of SIRT3-dependent aging driver tissues: liver, adipose tissue, endothelium, bone-marrow macrophages, and ovary—whose mitochondrial decline promotes inter-organ senescence signaling.
− Loss of SIRT3 activates ROS-sensitive transcription, SASP factors, and mitochondrial-derived vesicle release, thereby coupling mitochondrial stress to extracellular and systemic inflammatory circuits.
− Strategies such as NAD^+^ repletion, SIRT3 activation, and mitochondrial-targeted antioxidants hold promise for restoring mitochondrial quality and dampening chronic inflammation in aging-related disorders.
− Future studies should combine multi-tissue, sex-balanced models, longitudinal metabolomic profiling, and EV-based biomarkers of SIRT3 activity to test causal roles of mitochondrial signaling in organismal aging.

## Figures and Tables

**Table 1:** Sex-related differences observed upon SIRT3 loss and functional outcomes across major tissues.

TISSUE/SYSTEM	SEX-RELATED PHENOTYPE	PROPOSED MECHANISMS	REFERENCES
**KIDNEY**	**Male**-**biased** vulnerability to ischemia–reperfusion injury and oxidative damage	Hormonal modulation of SIRT3 determines mitochondrial resilience; overexpression rescues males	Shen et al., 2021, Belužić et al., 2023, Yao et al., 2024 [134,139,140]
**LIVER**	**Male**-**biased** metabolic impairment under high-fat diet; females retain protection unless estrogen-deprived	Metabolic stress reveals sex-dependent regulation of oxidative balance; estrogen sustains hepatic SIRT3 signaling	Pinterić et al., 2020, 2021 [148,149]
**BRAIN**/**NEURONS**	**Female**-**biased** vulnerability— impaired mitochondrial metabolism, redox balance, and cognition	Intrinsic (hormone-independent) dimorphism in neuronal mitochondrial regulation; possible X-linked or epigenetic factors	Pearson-Smith et al., 2023 [147]
**CARDIOVASCULAR** (**ENDOTHELIUM**)	**Female**-**biased** vulnerability: hypertension, oxidative stress, endothelial dysfunction	SIRT3 maintains nitric oxide signaling and mitochondrial homeostasis	Zeng, He, and Chen, 2020 [150]
**BONE**/**SKELETAL SYSTEM**	**Female**-**biased bone loss** mitigated by SIRT3 deletion	SIRT3 promotes osteoclast mitochondrial activity and resorption under estrogen withdrawal; inhibition protective post-ovariectomy	Ling et al., 2021 [15]
**REPRODUCTIVE ORGANS**	**Female**-**biased vulnerability:** ovarian aging accelerated by SIRT3 loss; testes largely unaffected	Oocyte mitochondrial dysfunction and oxidative stress drive premature ovarian decline; sperm development resilient	Zhu et al., 2022 [14]
**IMMUNE CELLS** (**MACROPHAGES**)	**Female**-**specific mitochondrial activation** and anti-inflammatory response	Estradiol-induced SIRT3 enhances deacetylase activity, lowers ROS, and reduces M1 inflammation	Barcena et al., 2024 [129]

## Data Availability

No new data were created or analyzed in this study. Data sharing is not applicable to this article.
